# DIY productive failure: boosting performance in a large undergraduate biology course

**DOI:** 10.1038/s41539-019-0040-6

**Published:** 2019-03-12

**Authors:** Sunita G. Chowrira, Karen M. Smith, Patrick J. Dubois, Ido Roll

**Affiliations:** University of British Columbia, Vancouver, BC V6T 1Z4 Canada

## Abstract

Students in first-year university courses often focus on mimicking application of taught procedures and fail to gain adequate conceptual understanding. One potential approach to support meaningful learning is Productive Failure (PF). In PF, the conventional instruction process is reversed so that learners attempt to solve challenging problems ahead of receiving explicit instruction. While students often fail to produce satisfactory solutions (hence “Failure”), these attempts help learners encode key features and learn better from subsequent instruction (hence “Productive”). Effectiveness of PF was shown mainly in the context of statistical and intuitive concepts, and lessons that are designed and taught by learning scientists. We describe a quasi-experiment that evaluates the impact of PF in a large-enrollment introductory university-level biology course when designed and implemented by the course instructors. One course-section (295 students) learned two topics using PF; another section (279 students) learned the same topics using an active learning approach, which is the standard in this course. Performance was assessed on the subsequent midterm exam, after all students had ample opportunities for practice and feedback, and after some time has elapsed. PF students scored nearly five percentage-points higher on the relevant topics in the subsequent midterm exam. The effect was especially strong for low-performing students. Improvement on the final exam was only visible for low-performing students. We describe the intervention and its potential to transform large introductory university courses.

## Biology Education: Early failure supports future learning

While first-year biology students failed to answer conceptual questions on their own, these experiences helped them learn better. Sunita Chowrira and colleagues at the University of British Columbia took a different approach to teaching cell-biology topics. In their classroom, students first struggled to solve complex problems, which primed them to learn the expert solution via a series of interactive activities - activating prior knowledge, then sifting through the available information, to arrive at a solution. This approach, termed Productive Failure, led to significant benefits compared with the more conventional instruction. The Productive Failure approach was effective especially among lower-performing students, for whom benefits in learning persisted also into the final course exam. These results suggest that applying Productive Failure in higher education science courses may support all learners while narrowing the achievement gap.

## Introduction

Students beginning their university education often express the sentiment that first-year science is hard. One reason for the difficulty is that students are now expected to apply and problem-solve using the factual information they have amassed.^1–3^ While students may have managed to get through secondary school and into university successfully using ineffective surface learning approaches,^4–6^ these approaches are often not enough to succeed in university courses. Biology, the largest undergraduate program in the Faculty of Science at the University of British Columbia, has its share of students struggling with conceptual understanding of the fundamental concepts that form the foundations for more advanced topics. To better prepare our students, we need to provide ample opportunities that promote conceptual knowledge. Rittle-Johnson et al.^7^ define conceptual knowledge as “understanding of the principles that govern a domain and of the interrelations between units of knowledge in a domain.” It requires students to synthesize new knowledge with existing knowledge, leading to robust mental models that support transfer of knowledge and flexible application in areas other than that in which it was learned.

Teaching that supports problem-solving and achieves conceptual understanding has been identified as a complex endeavor. Incorporating active learning strategies, where students are provided the opportunity to apply concepts as they learn them, has been documented to be effective in promoting learning.^8–11^ Most innovative approaches to Biology teaching in recent years are based on a constructivist framework,^12–16^ where the classroom instructions are structured around active learning approaches with the intention of maximizing student learning.^17^

Successful implementation of active learning is not obvious and requires intricate classroom choreography.^18^ It was found to be especially challenging in large classrooms.^17^ Issues generally inherent to classrooms in post-secondary institutions include inadequate pre-class preparation, inactive or non-responsive behaviors, discomfort with failure, and lack of motivation or perseverance.

One pedagogy that is rarely used in higher education is productive failure (PF). In PF, learners attempt to solve challenging problems prior to receiving explicit instruction on the target domain.^19^ While learners often fail to succeed in the task itself, it prepares them for meaningful future learning.^20–22^

Several factors convinced us to evaluate the impact of PF. First, PF focuses on conceptual understanding, future learning, and transferability to new topics,^19–23^ which are all challenging tasks for students in first-year university. Second, PF seems to address other important aspects of learning at post-secondary institutions, such as fear of failing,^24–26^ and recovering from failure. It is important to note that PF is a form of extreme active learning, and active learning is typical for these classes. Learners work in small groups and construct shared solutions by building on prior knowledge and making sense of the challenges at hand,^27^ techniques that fall under the highest level of active learning, ‘Interactive’’, as defined by Chi and Wylie.^11^ Could PF prove effective even compared to standard best practice?

This study was conducted in a large-enrollment first-year Cell-Biology course that is taken by thousands of students every year, mostly from the Faculty of Science. Other students typically come from Applied Science and Land and Food Systems. Based on several years of performance data on exam questions that target conceptual understanding, we identified key topic areas where students consistently underperform. While most students perform well on assessment questions at the lower Bloom’s cognitive levels^28^ (e.g., define the process or list the steps involved), many struggle with questions at the higher levels of Bloom’s taxonomy (e.g., predict the outcome or interpret the reasons for the observed data). Diagnostic use of an in-house concept inventory (CI), the Transcription and Translation CI,^29^ further highlighted the prevalent misconceptions in topic areas of replication of biological information (DNA replication), and biological information flow (Transcription and Translation). Concept Inventories are validated testing instruments,^30,31^ used routinely to identify alternate conceptions or misconceptions among students. They evaluate conceptual knowledge by focusing on meaning making and relationships between concepts, rather than procedural problem-solving. In the target course, the Transcription and Translation CI helped identify the fundamental concepts underlying the two topic areas that we then chose for the PF study: DNA replication and Transcription and Translation. The identified concepts in these two topic areas that students struggle with, include the structural directionality of the genetic material, functional directionality of the process of Transcription and Translation, and the context of the consensus regions within the genetic sequence that dictate the accuracy, feasibility and fidelity of the start and stop of the processes.

The target course has several sections that are typically taught using shared materials and approaches (even if by different instructors). Every week, students are assigned a pre-reading and a pre-reading quiz, to introduce students to the topic and the relevant terms ahead of class. For the two topics mentioned above, we compared the PF classroom approach to the active learning (AL) approach, and implemented them in two of the sections in the course as described below. The instructor of one of these sections was interested in evaluating PF in that section; a second instructor agreed to share data from their section as a comparison group. All students in these sections, who attended class during the activities and the assessments, were included in the study.

## PF implementation

**Fig. 1:**
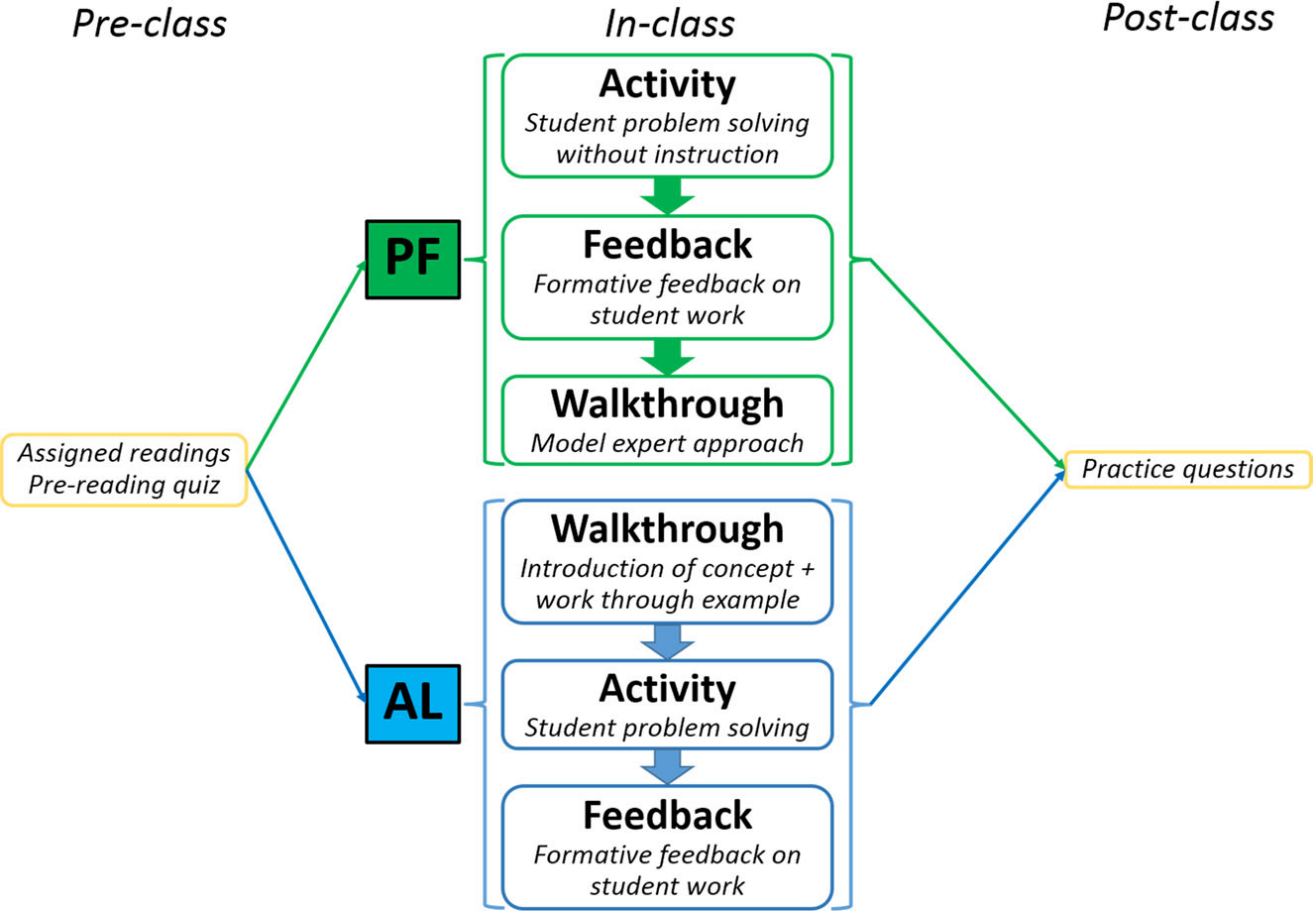
Flow-chart of active learning (AL) and productive failure (PF) implementation in the classroom for different sections

As done every week, students were asked to read the relevant chapter prior to class and complete the pre-reading quiz. Class time was modeled after the PF cycle^32^ and contained two main phases, an Exploration phase and a Consolidation phase. The first part of the class time (~25 min), the Exploration phase, was devoted to student problem-solving activity in small groups (see Fig. 1). The activities were designed at higher cognitive levels of Bloom’s taxonomy, involving data interpretations, outcome predictions, and deliberately designed to be challenging. During the problem-solving activity phase, students worked in groups of 2 to 4, with minimal or no introduction to the topics other than the previously assigned pre-reading for the class. Student responses were collected at the end of the activity. This was followed by the Consolidation phase. First, students were provided with formative feedback (~5 min, immediately following the activity). During this part, the student responses collected from the activity were used as options for a set of classroom-response (aka “Clicker”) questions. This enabled the instructor to provide immediate formative feedback on example work produced by the students. Remaining class time was devoted to a Walkthrough (~20 min), to close the learning loop. During this part, the instructor modeled an expert’s way of approaching the activity, while continuing to involve students in this process by prompting and eliciting their contribution to the problem-solving path.

## AL implementation

Similar to the PF condition, students in AL condition were asked to read the relevant chapter prior to class, and to complete the pre-reading quiz. The first part of the class time was used by the instructor to introduce the concept (Walkthrough; lower part of Fig. 1), and work through an example problem related to the concept, interleaved with Clicker questions (~25–30 min. of class time). Prior to responding to the Clicker questions, students were given ample opportunities to actively engage with the materials being presented, in the form of group discussions. The remaining part of the class time (~20–25 min.) was devoted to practicing problem-solving, where students worked in groups to solve problems similar to the example seen during the first part of the class, and received formative feedback on their work. The problems were similar to the ones used with the PF group: designed at higher cognitive levels of Bloom’s taxonomy, involving data interpretations, outcome predictions, and challenging.

**Fig. 2:**
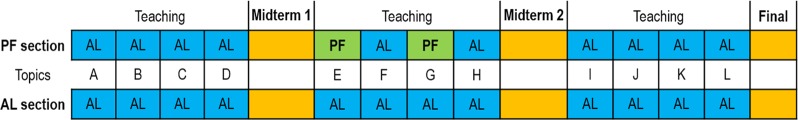
Parallel structure of sections, showing variation in teaching technique (active learning (AL) vs. productive failure (PF)) for two topics

Notably, all other topics in the course were implemented using a similar approach to the AL instruction described above (see Fig. 2). While the sections had different instructors, the teaching team collaborated on shared resources and administered common exams.

The implementation described above differs from most previous PF studies in several important aspects. First, this study is in the life sciences domain. Many PF studies to date have taken place in the domain of statistics. The use of statistics focuses on topics in which intuition can play a role, such as variance. This creates an affordance to evaluate students’ invented methods, identify their shortcomings, and learn from their failures. Exploratory PF studies in process engineering,^33^ climate science,^34^ physics,^35^ and math,^36^ offer similar insights. Loibl et al.^22^ call for evaluation of PF in broader domains, as done here. It is unclear whether PF works in a topic that requires more expertize and offers fewer opportunities for using one’s intuition and life experiences as baseline for evaluation.

Another contextual difference from most prior literature is the population. Most PF studies to date were implemented with K-12 learners.^22^ In contrast, the current study was implemented with university students. We have identified a few studies that evaluated PF with university students,^33,36–39^ however, none of these were in a typical life sciences classroom setting. Implementing PF in this context has unique challenges. First, instructors and students alike may perceive the classic lecture to be efficient and sufficient. Further, they may perceive students to be expert learners who do not need to invest time in complex designs such as PF. From an attitudinal perspective, university students may have worse dispositions towards failure, as, simply put, they are not used to failing. Altogether with high anxiety caused by the transition from high-school to university, PF may be perceived to have detrimental effects on their learning.

A third difference is our use of pre-reading assignments to pre-expose students to the target concepts. While this aspect of PF differs from other common implementations of PF (cf. Loibl et al.^22^), it builds on common practices in science classes in higher education. Students often come to class underprepared. Targeted pre-reading assignments, which were used here, support students in activating relevant prior knowledge and acquiring basic vocabulary to be elaborated and built upon during class time.^40^ Indeed, these are also preconditions of successful PF activities.^32^ The high pace of college classes, as well as the specificity of their vocabulary, introduced a need to expose students to the language in order to allow them to construct meaningful (albeit wrong or partial) solutions. The pre-reading assignment was incorporated to help students come prepared to the class activity. Thus, while the practice of pre-reading is not common to PF studies, it helped us adhere to the principles behind PF. Notably, students in both conditions received the same pre-reading assignment. In both cases, it is perceived as a preparatory activity and does not replace classroom activities or lectures.

Yet another important aspect of our implementation is that of the non-PF comparison condition. Students in the comparison group of the present study received highly effective active learning instruction that had been refined over several years, as supported by the Science Education Initiative at the university.^18^ AL in higher education means that rather than following a lengthy lecture with a duration of practice, practice and support are intertwined and interleaved, and short instructional periods build upon matching activities (for instance, using worksheets and Clickers). Thus, students in this study’s comparison condition received what is believed to be better instruction than passively listening to a lecture (often referred to as Direct Instruction^11^). We found very few studies that contrasted PF with a similar form of AL. Several studies have contrasted design features within PF (cf., Loibl and Rummel^41^ for PF with or without contrasting cases; Kapur,^42^ Kapur and Bielaczyc,^43^ and Roll et al.^44^ for the need for design as part of PF; and Roll and colleagues^23,45^ for metacognitive support given during PF). However, as far as we know, only one study (Kapur^46^) contrasted PF with AL that provides active feedback and guidance to learners. In that study, Kapur^46^ compared learning from PF with learning from similar activities in which the instructor actively guides the students through the process, providing feedback, and highlighting conceptual errors. Kapur found that students who struggled in the PF condition outperformed their peers who received more support and were guided through their problem-solving. Here, we seek to evaluate a similar comparison in another context. It may be that part of the success of PF has to do with facilitating more active learning compared with the passive Direct Instruction.^11,16^ The current study compares two forms of active learning in post-secondary Life Sciences classrooms.

Perhaps the most important difference between this study and previous studies is the role of the researcher. Previously, most studies were designed by researchers who are familiar with the theory behind PF. Being a complex topic that requires disciplinary expertise, instruction in the present study was designed and implemented by the course instructors, and the fidelity of PF implementation was achieved through an iterative process. Implementation by instructors (disciplinary experts) is an important hurdle that novel pedagogies should clear before they can be considered relevant to a wider audience. From an ecological validity perspective, do students benefit from PF when designed by the course instructor and compared against good classroom teaching? From an external validity perspective, what are the boundaries of PF—specifically, does it produce the expected results also in the context of post-secondary biology learning? Thus, overall, our research question in this study is one of scalability and validity.

## Results

### Sample characteristics

We gathered complete course data for 574 students: 295 in the PF condition, and 279 in the AL condition. Overall, 32% self-identified as male, and 96% were in their first or second year of university. No significant relationship was found between university year and grades, but those enrolled in the Faculty of Science showed a general advantage in grades, of 15.95 ***p < 0.001, 95% CI = [9.25, 22.64], *t*(391.98) = 4.68; *d* = 0.42.

Between conditions, there were no significant differences by gender or university year, but students in the Faculty of Science were more likely to be in the PF condition, *χ*^2^(1, *N* = 574) = 10.85, *p* < 0.001. This difference was coincidental and went unnoticed until analysis of study data. Possibly due to differences in sample composition, significant differences (by condition) were observed in regular course exam marks (unrelated to the study). On those marks, students in the PF condition outperformed their counterparts on Midterm 1 (5.14 ***p < 0.001, 95% CI = [3.23, 7.06], *t*(559.31) = 5.28; *d* = 0.44) and final exam (7.63 ***p < 0.001, 95% CI = [4.75, 10.52], *t*(567.02) = 5.20; *d* = 0.44), but not on Midterm 2, (−1.51, *p* = 0.23, 95% CI = [-4.01, 0.98], *t*(561.03) = −1.19; *d* = −0.10), as shown in Table 1. In order to account for these differences, performance on these “other’’ items served as covariates in our model.

**Table 1:** Raw exam scores, by condition, showing mean (standard deviation)

		Study items		Other items		
Condition	*N*	Midterm 2	Final	Midterm 1	Midterm 2	Final
AL (active learning)	279	67.96 (19.81)	66.09 (28.11)	76.23 (12.18)	73.50 (15.82)	64.15 (17.89)
PF (productive failure)	295	73.97 (19.35)	74.31 (25.13)	81.38 (11.08)	71.98 (14.54)	71.78 (17.22)

### Effects of condition on learning

**Fig. 3:**
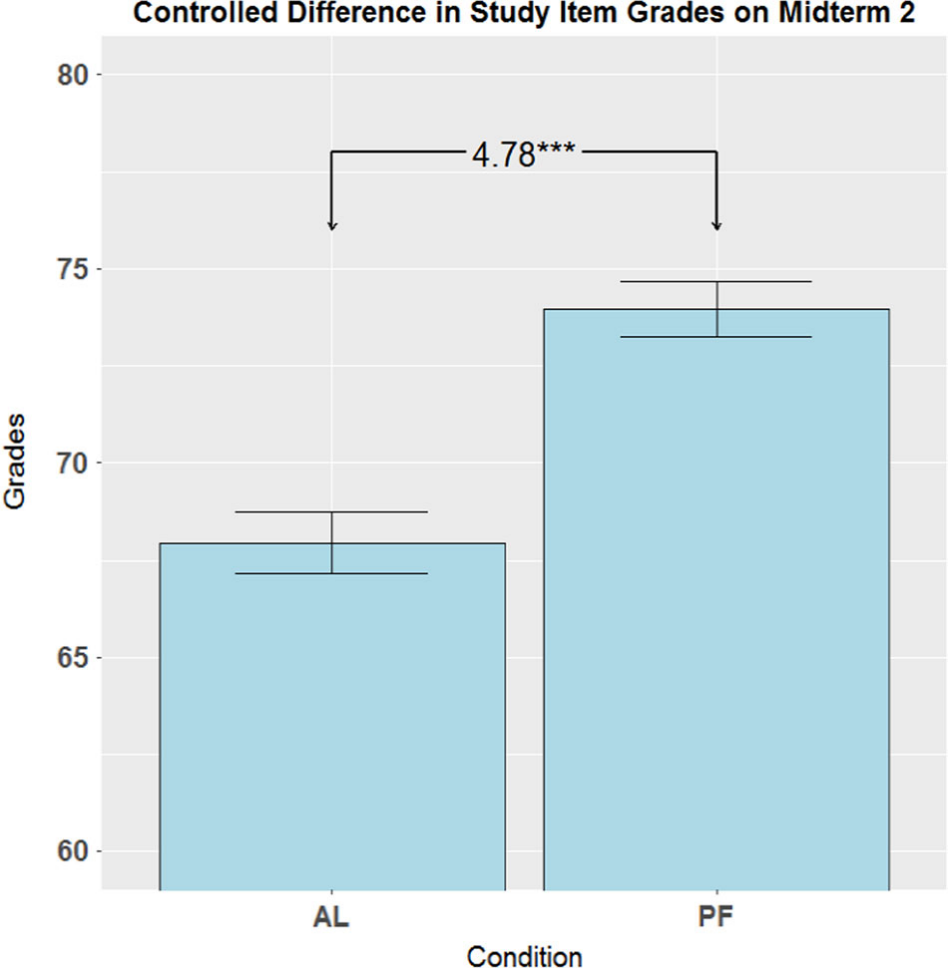
Adjusted mean study item grades (0–100) and Standard Error by Condition, controlling for gender, university year, program, Midterm 1 grades and Midterm 2 non-study items. Error bars show standard error. ***p < .001

Student performance on the midterm and final exams was analyzed, blind to condition. The differences described above were not unexpected for a quasi-experimental student sample. To isolate the effects of learning condition on student performance, we used linear regression to predict scores (0–100) on topics where teaching approach varied, controlling for scores on Midterm 1, gender, university year, enrollment in the science program, and exam scores for questions in the same exam on topics where teaching was not varied (referred to as “other items”). Thus, differences between student populations or instructors, as reflected by performance on other items, were accounted for, isolating the impact of PF vs. AL on the studied topics. For Midterm 2, (following the implementation of PF) the resulting coefficient for the PF condition was significant, *b* = 4.78, 95% CI [2.19, 7.36], *t*(567) = 3.63, *p* < 0.001, with an effect size of 0.32, 95% CI [0.15, 0.49] (derived by dividing the coefficient by the residual standard error). This shows that students in the PF condition had grades for those topics almost five percentage-points higher (Fig. 3). The overall model fit was *R*^2 ^= 0.43, *F*(6,567) = 71.95, *p* < 0.001, which means that 43% of those condition-topic scores could be explained by this model. To account for the large number of covariates, we ran regression models with stepwise variable selection. Results were nearly identical to those reported above, and thus we focus on the more straightforward, comprehensible, and complete model.

A similar analysis was conducted for the final exam, but condition effects were much smaller and failed to reach statistical significance, with the difference in topic grades (on the final exam, controlled for non-topic scores on the final) being *b* = 0.78, 95% CI [−2.80, 4.36], *t*(567) = 0.43, *p* = 0.668.

### Condition effects by student ability

Next, we evaluated the dependency of this effect on student ability. We used Midterm 1, which took place before the manipulation, as a proxy of student ability. We then split our sample into three parts by ability, with the lower being at or below the 33rd percentile (a grade of 75%) and the upper being at or above the 67th percentile (a grade of 85%).

The sub-sample sizes and resulting regression coefficients for the PF condition (representing difference in topic marks for Midterm 2, controlled as above) are shown in Table 2. For Low-achieving students, the effect of PF is the largest. In fact, as seen in the *b* value, low-achieving students in the PF group scored eight points higher than their counterparts in the AL group, even after controlling for gender, faculty, year, and performance on other items. This effect is considerable for that group.

**Table 2:** Productive failure (PF) increase on Midterm 2 by ternary split of ability

Ability	*N*_PF_ + *N*_AL_ = *N*	PF increase
Low	84 + 110 = 194	*b* = 8.28, 95% CI [3.68, 12.89], *t*(187) = 3.55, *p* < 0.001
Med	92 + 97 = 189	*b* = 3.26, 95% CI [−1.67, 8.18], *t*(182) = 1.31, *p* = 0.193
High	119 + 72 = 191	*b* = 3.37, 95% CI [−0.71, 7.45], *t*(184) = 1.63, *p* = 0.105

The effect of PF on Med and High achieving students does not reach significance, likely because splitting the population reduces statistical power. As the coefficients for Med and High were similar, we examined the combined data for these two groups. The coefficient for the combined group reaches significance, *b* = 3.25, 95% CI [0.07, 6.43], *t*(373) = 2.01, *p* = 0.045. This suggests that the PF technique benefitted the lower-performing students more than twice as much, yet still had a positive effect on higher performing students.

A similar split pattern emerged for effects on final exam scores. The effect of condition was marginally significant for lower-performing students, where PF outperformed AL by almost seven points. This effect did not reach significance for high performing students (Table 3).

**Table 3:** Productive failure (PF) increase on Final exam by ternary split of ability

Ability	PF increase
Low	*b* = 6.83, 95% CI [–0.29, 13.94], *t*(187) = 1.89, *p* = 0.060
Med	*b* = –3.87, 95% CI [–10.19, 2.45], *t*(182) = –1.21, *p* = 0.229
High	*b* = 0.55, 95% CI [–4.79, 5.88], *t*(184) = 0.20, *p* = 0.840

## Discussion

Our quasi-experiment showed that PF instruction yields better performance, compared with the conventional AL approach, on the exam following the manipulation (Midterm 2). This trend persisted for low-performing students also on the final exam, but was virtually eliminated for better performing students.

As commonly observed in quasi-experiments, differences between conditions existed in the outset, as shown for Midterm 1 and the other items on the Final exam (due to sample characteristics, instructor, or a combination of factors). As we control for performance on these items, the benefit of PF on manipulated items needed to be much larger in order to surpass other differences. Thus, the results presented here are a conservative estimate of the true value in PF.

Notably, students had considerable exposure to study topics following study and before the test. All students received practice problems, and all had time to prepare for Midterm 2, with several weeks between manipulation and testing. Thus, the effect of PF instruction in this study was long-term and fairly robust, compared to previous PF studies involving immediate post-tests. In fact, the effect of PF on low-achieving students also carried forward to the final exam, as discussed further below.

While PF instruction proved fruitful in Midterm 2, the effect was much smaller by the final exam. There are several potential explanations for this. It may be that the condition-topic questions on the final exam were too few and/or did not capture ability as well as the Midterm 2 condition-topic questions. An alternative explanation is one of decay, that is, the PF effect simply faded over time. A third likely explanation has to do with exposure. All students had ample opportunities to practice these concepts in subsequent tutorials, and thus bring their knowledge to a similar level regardless of initial instruction. The most likely explanation is that, being a final exam, students study for it and strategically focus their attention on topics that they know less. Thus, differences that might have existed between conditions were all but eliminated by students’ independent learning. The fact that students who performed lower on Midterm 1 still showed effect for PF also on the Final exam may strengthen this explanation, as these students are evidently less successful learners in this course. Further research on temporal aspects of PF, as well as its interaction with student ability, is warranted.

Our results suggest that students who are more challenged in the course (as reflected in Midterm 1) benefited more from PF, on both Midterm 2 and the Final exam. One possible explanation is that learning from instruction is challenging and may be cognitively overloading. Conceptual learning requires understanding of key concepts and how they fit together. To avoid cognitive overload during class time, all students were assigned pre-reading in our study. It may be that pre-reading alone was not sufficient for low-performing students. Instead, the addition of hands-on experiences helped them acquire basic tools with which they could make meaning of subsequent instruction. Schwartz et al.^27^ describe the benefits of these expository activities to help students construct relevant (prior) knowledge that assist them in learning from future instruction. Similar results, in which lower-performing students benefit from exploring the solution space, were also found in Geometry problem-solving^47^ and Physics simulations.^48^ It is important to emphasize, though, that these results are contingent on using Midterm 1 as a proxy for student ability. Further research is warranted to evaluate the robustness of this pattern.

Given that students in both conditions received support for active learning, this aspect of PF is probably not to be attributed for its success. If so, why did PF students outperform the AL students? When explaining the effect of PF, Loibl et al.^22^ build on Kapur and Bielaczyc^32^ to identify the following relevant mechanisms: prior knowledge activation, awareness of knowledge gaps, and recognition of deep features. Active learning also seeks to activate many of these mechanisms. Frequent classroom-response (Clicker) questions encourage students to activate their prior knowledge, and provide feedback to support awareness of knowledge gaps. We attribute the difference in learning between conditions in our study to two factors: depth of search and availability of solution.

By depth of search we refer to students’ struggle to develop a model that can solve the problems they are given. The quick turnaround of classroom-response questions provides students with feedback, but does not provide them with enough time to fully understand the requirements of the problems (and the limitations of their own solutions). This may lead to a difference in the third mechanism identified by Loibl and colleagues, recognition of deep features. In addition, there is some evidence that self-generated feedback (as done in the PF condition during the first phase) may lead to better learning compared to only receiving external feedback.^49,50^

The second significant factor is the availability of the canonical solution in the AL condition. Having a solution may encourage students to accept it “at face value”, without dissecting its deep structure, and without fully grasping its deep features.^32,37,51^

Our research question focused on ecological validity. Is PF superior to AL in situations in which instructors create their own version, in relatively complex (and less intuitive) topics?

Our Biology PF activity design (Fig. 1) was based on the design guidelines from Kapur and Bielaczyc.^32^ For the “Exploration” Phase, we developed problems that required students to access and elicit prior knowledge, apply it in a novel context, seek out appropriate resources, and generate and explore solutions with minimal assistance from the teaching team. The problems were designed to be challenging but not frustrating. In the topic area of Transcription and Translation, for example, students were required to access prior knowledge (from assigned pre-reading or from what they learned in high-school Biology courses) about the steps involved in the processes, the various structural parts needed for the processes (requiring reasoning across orders of magnitude – atomic to systems), the roles of the structural parts, and the sequence in which the roles occurred (requiring reasoning across ontological levels—e.g., DNA is information, a unit of inheritance, and a physical entity^52^). During the Exploration phase of the PF activity, accessing prior knowledge alone was not sufficient. Students also had to use the accessed knowledge appropriately by asking relevant questions necessary for generating the solutions on their own. This problem-solving phase provided opportunities for students to explore the affordances and constraints of multiple solutions.^20^ During this phase of struggle, modest support was provided by the facilitating teaching team in the classroom to help activate metacognitive processes and prevent onset of the frustration phase. Support was given in the form of prompts to focus students’ attention without providing additional information, such as, “what information do you have in the problem?”; “what other kind of information would you need in order to continue the process?”; or “where might you find this information?.” This support helped students understand the challenges and make progress, yet they mostly failed to construct complete and correct solutions.

The Consolidation phase of the PF activity (our post-activity Formative Feedback and Walkthrough phases) built on the solutions produced by the students in Activity phase. The student solutions, containing their mistakes, misconceptions, or gaps in knowledge, were used as examples by the instructor to provide feedback and to walkthrough the problems, in a logical, guided, and expert-like manner. The consolidation phase provided the students opportunities for comparing and contrasting, organizing, and assembling the relevant student-generated solutions into appropriate solutions.^20^

The study described above has several limitations. First and foremost, our choice to focus on ecological validity limited our ability to conduct a randomized controlled trial. Instead, this was a quasi-experiment; both sections were taught by different instructors, and students self-selected into sections based on schedule and other factors. For example, unexpectedly, the PF group had a higher ratio of students from the Faculty of Science, who are traditionally more successful in this course. Consequently, they performed better on some measures of knowledge. We compensate for these variations statistically, by (i) controlling for performance on a prior exam, (ii) controlling for person variables of enrollment in the faculty of science, university year and gender, and (iii) adding other items from the studied tests to our measures. However, such variation may lead to indirect impact, such as class dynamics. The fact that the results of this quasi-experiment echo earlier results (cf. Kapur^46^), suggests that sample characteristics are not the main contributing factor.

Another limitation is the fact that only one instructor taught using PF. As the study topics are highly discipline-specific, instruction was designed solely by the instructor. However, this was done with advice from a learning scientist, who provided motivation, relevant literature, design principles, and general emphases for the activity. This support however, was not specific to the two activities, and feedback directly on the activities was not provided. As the paper argues for a “Do-it-Yourself” (DIY) approach for PF, future work will need to show how well this transfers to additional instructors with even less support.

Lastly, our analysis by student performance relies on Midterm 1. This is a single exam of limited scope, early in the term, and thus these results are more suggestive than conclusive. Further study into aptitude-treatment interaction are warranted.

Overall, results show that PF instruction helped all learners achieve greater gains on a subsequent exam, even after several weeks had passed and all students received additional exposure to these topics. Results were especially positive for low-performing students, for whom the benefits of PF instruction lead to improvement of nearly seven points on relevant topics in the final exam of the course. These results show the promise and potential of PF in first-year large-enrollment university courses when implemented by the course instructor, and also as compared with the use of alternative effective active learning techniques.

## Methods

As the goal of this research was to replicate PF studies with high ecological validity, a quasi-experimental approach was taken. Two simultaneous sections of an undergraduate introductory biology course were chosen as comparison groups, having similar numbers of students, equivalent curricula, and shared materials. These two sections had identical syllabi, textbooks, content order and exams: two midterms and a final. Only two topics of the course were explicitly varied in teaching technique; one section had those topics presented using AL techniques (like the rest of the course), while the other section had the topics (and only those) presented using PF. This difference in technique (our “condition”) was implemented after Midterm 1, on topics that were evaluated (among others) during the second midterm, Midterm 2 (see Fig. 2). Midterm 2 consisted of 27 multiple-choice questions, of which ten were about the condition topics (Cronbach *α* for study items = 0.574). The final exam consisted of 47 multiple-choice questions, of which five were about the condition topics (Cronbach *α* for study items = 0.555).

The two topic areas—replication of biological information (DNA replication), and biological information flow (Transcription and Translation)—are complex key processes in cell biology. A broader understanding of a number of other cellular processes can be linked to grasping the concepts embedded in these topics. In each topic area, there are many cellular parts (large and small molecules) at various steps; so that understanding structure, function, and order is important. A common approach in a traditional instructional design would introduce each step, the parts in the process, and their related functions. Students could then be assessed on each step and the overall product. The problem with this approach is that students mainly memorize each step, the different parts and their functions, rather than attempt to have a deeper understanding of how they might all fit together, how their specific roles might affect their functions downstream, and how these might then relate to other processes.

Our intent overall in this course was to steer students away from procedural learning, and instead, have them work through the process conceptually. Recall that, prior to each class, students in both AL and PF conditions were assigned pre-class readings and quizzes. The implementation difference in the two conditions is described below:

The PF instructional approach (see Fig. 1) consisted of three phases:

- *Activity* phase (the PF Exploration phase): Students were tasked with working through a problem in small groups. The PF problems were designed to be open-ended (e.g., show possible products when the given stretch of DNA sequence might be transcribed and translated within a cell), so that when given a starting point (e.g., a DNA sequence) students would design the process to mimic a cell to yield the end products. This required students to think deeply about what was needed (parts), their roles (function), their order, and the process itself. During the first iteration of PF implementation, many students found the task to be too difficult and gave up on the activity out of sheer frustration. Taking note of this, PF implementation was refined to include “moderate support” during subsequent iterations, to reach the sweet spot where failure did not necessarily lead to students giving up entirely. The challenge that students encountered was conceptual. Students were aware of the general principles related to the process of Transcription and Translation. However, the task required applying these in the cellular context. This context has its own constraints and affordances that define the Transcription and Translation process. Understanding how the process unfolds in the cell was the challenging component. Specifically, this is a very precise process, and thus students needed to construct an accurate mental model of what is happening in the cell. The “moderate support” provided looked something like this: when students got stuck, they were asked to describe what information they had and what other kind of information they would need in order to continue the process, and where they might find the information, i.e., activating metacognitive processes. With the Teaching Assistants providing such occasional modest hints to help students get “unstuck”, inevitably one or more student(s) in the group would have an “aha moment” and help the rest in the group move past the sticking point. Support helped students understand the challenge and move forward, but still the final output produced by most were inadequate and/or inaccurate.
- Formative *Feedback* (the PF Consolidation phase) on student work (Fig. 1): At the end of the activity, completed student responses were collected by the teaching staff. Although the collected responses were not marked for correctness, the student material provided the teaching team the opportunity to quickly review roughly what proportion of the class were able to complete the activity, and where the students struggled the most. The student responses in the collected activity sheets also served as an excellent source of alternative answers for the feedback Clicker activity. Occasionally, example responses from previous iterations of the classes were used.
- *Walkthrough* (the PF Consolidation phase): Seeing their mistakes and the reasons for them during the formative feedback primed the students for the Walkthrough phase, during which the expert’s approach to solving the problem was modeled by the Instructor. This process was essentially an interactive use of a worked-out example,^53^ where students were encouraged to contribute to the walkthrough.

In the AL instructional approach (see lower part of Fig. 1), class time was divided into the following three components:

- *Walkthrough* phase: Short instructional periods, interspersed with student engagement components (e.g., “think-pair-share” or group discussion before answering Clicker questions), was used to introduce the topic and work through an example problem to highlight the underlying concepts.
- *Activity* phase: Students worked through a problem similar to the example problem seen during the walkthrough phase, in groups. Students in general, worked through the problems without requiring much assistance from the teaching team. At the end of the activity, student responses were collected.
- Formative *Feedback*: The collected student responses were used as examples to point out misconceptions or missing components, or the extent of completeness, or they were used as distractors for Clicker questions.

All exams (midterms and final) were common to both the groups in the study. The exam questions were designed to span the Bloom’s taxonomy. While a few basic process/step questions required recall, many questions on the exams required students to work through the steps, both forwards and backwards, thus requiring application of the learned concepts. A few questions presented a variation of the process that required analysis and synthesis. For example, students were asked to predict how a process might be impacted by a mutation or to predict the outcome with the addition of a new molecule to the system.

The three exams (all multiple-choice) were administered as typical course exams, with no special procedures related to this study. At no point were students told they were being given a deliberate variation in educational technique. Given that students typically experience a variety of teaching approaches, and instructor personalities and values, it was expected that, should any student confer with another from the other section, any noted difference in technique could easily be attributed to the normal variation students encounter when having different classes and instructors. Thus, we expected no demand characteristics of our condition, i.e., not knowing what we were up to, students would not react to it.

All exams were scored in the typical way, as the mean of question scores for each student, framed herein as percentage correct (0–100). Due to the small number of test items related to the two topics, and to maintain a scale that is sufficiently consistent, questions for the two condition topics were combined as one condition score for Midterm 2 and one for the Final.

To statistically isolate condition effects from across-section effects, we used a simple linear regression model, predicting condition topic scores (0–100) from a dummy variable for condition (PF = 1, AL = 0) plus gender (male/female), university year (1–4), program (science or other), Midterm 1 scores (0–100) and same-exam scores (0–100) on other topics as control variables. The regression coefficient for this dummy variable then represents the effect condition had on topic scores, controlling for the other variables. A linear regression model was chosen since it is robust and makes fewer assumptions regarding the data.

Methods were approved by the UBC Behavioral Research Ethics Board (UBC BREB #: H14-02217). Methods were performed in accordance with relevant regulations and guidelines. As part of the procedure, all students consented for their data to be included in the study. They had the opportunity to opt-out from the study, and none chose to do so.

## Data Availability

The datasets generated during and/or analyzed during the current study are available from the corresponding author on reasonable request.
